# Endoscopic resection of knosp grade 0–2 growth hormone-secreting pituitary adenomas with routine medial cavernous sinus wall excision

**DOI:** 10.1007/s00701-026-06946-9

**Published:** 2026-06-11

**Authors:** Peter Bill Juul Ladegaard, Marianne Skovsager Andersen, Peter Darling, Morten Winkler Møller, Jeanette Krogh Petersen, Christian Bonde Pedersen, Frantz Rom Poulsen, Eric Wang, Bo Halle

**Affiliations:** 1 Department of Otolaryngology, Head and Neck Surgery, Odense University Hospital, Winsløwsvej 4, DK-5000 Odense, Denmark; 2 Department of Endocrinology, Odense University Hospital, Winsløwsvej 4, DK-5000 Odense, Denmark; 3 Department of Neurosurgery, Odense University Hospital, DK-5000 Odense, Denmark; 4 Clinical Institute and BRIDGE (Brain Research - Inter Disciplinary Guided Excellence), University of Southern Denmark, Odense, Denmark; 5 Department of Pathology, Odense University Hospital, DK-5000 Odense, Denmark; 6 Department of Otolaryngology-Head & Neck Surgery, University of Pittsburgh Medical Center, 208 Lothdrop Street, Pittsburgh, PA 15213 USA

**Keywords:** Pituitary, Adenoma, Resection, Cavernous sinus, Endoscopy

## Abstract

**Background:**

Endoscopic surgical resection is gold standard treatment for growth hormone-secreting pituitary adenomas (GH-PA). However, biochemical remission rates are only approximately 40–60% after adenoma resection. Emerging evidence suggests that invasion of the medial cavernous sinus wall may contribute to persistent disease, even in low Knosp grade GH-PAs. This single-center study evaluated clinical and biochemical outcome of GH-PA resection with medial cavernous sinus wall excision—an approach that has demonstrated promising results internationally. We compared these data with two historical cohorts from our own institution operated microscopically and endoscopically respectively.

**Methods:**

Fourteen patients had endoscopic GH-PA resection with medial cavernous sinus wall excision (ERA + MCS) at Odense University Hospital during 2023 to 2025. All procedures were performed by a specialized skull base surgical team. Patient outcomes were assessed based on radiologic and biochemical remission at 3 and 6 months and perioperative complications were evaluated. The historical cohorts comprised of 29 patients operated microscopically with adenoma resection (MRA) and 16 patients operated endoscopically with adenoma resection without excision of the medial cavernous sinus wall (ERA-MCS). In all three cohorts the surgeries were primary.

**Results:**

Endoscopic GH-PA resection with medial cavernous sinus wall excision resulted in complete 6 months biochemical and 3 months radiological remission in 86% of primary GH-secreting adenoma (Knosp grade 0–2). No intraoperative complications were observed, and early postoperative complications were minimal, with transient syndrome of inappropriate antidiuretic hormone secretion occurring in two patients (14%). Remission rates in the historic cohorts, were 31% (MRA) and 38% (ERA-MCS), respectively.

**Conclusion:**

Our data confirms that endoscopic resection of GH-PAs with routine medial cavernous sinus wall excision is a safe and effective surgical approach yielding high remission rates.

## Introduction

Pituitary adenomas (PA) account for 10–25% of all intracranial tumors. The incidence is 1/1100 [22]. Growth hormone secreting pituitary adenoma (GH-PA) is the second most frequent hormone producing PA (12 per 100.000) [1, 7, 18]. Based on tumor size, GH-PAs are classified as macroadenomas (≥ 10 mm) or microadenomas (< 10 mm). At the time of diagnosis, approximately 75% of GH-PAs are macroadenomas [8, 11]. The clinical manifestation of a GH-PA is acromegaly, characterized by macroglossia, excessive somatic growth, increased body mass index, mandibular and nasal overgrowth, enlargement of the hands and feet, and dental diastema, but patients may have few symptoms and the phenotype may not be obvious [1, 13]. Patients with untreated acromegaly have increased morbidity including different cancers (thyroid and colorectal), cardiovascular disease, osteoporosis, and sleep apnea [3, 18, 23]. In addition, 30% of GH-PA co-secrete prolactin [11]. The adenoma itself may also compress adjacent structures e.g. the pituitary gland causing hypopituitarism, the pituitary stalk causing hyperprolactinemia and the optic apparatus leading to visual impairment [1, 13].

Surgical resection is the gold standard for treating GH-PA. Surgical techniques have evolved considerably. For many years, microscopic transsphenoidal resection was the preferred method; however, the advancement of optical technology has since made endoscopic transsphenoidal approach the preferred method [13, 16]. Endoscopy has fewer complications, enabling more radical resections, and lower recurrence rates [16, 20]. However, recurrence remains a challenge, often necessitating adjuvant medical therapy, reoperation or radiotherapy. Biochemical remission rates for GH-PA endoscopic surgery are reported to be around 40–70% depending on adenoma size, extent of resection and whether it is a first-time surgery or reoperation [2, 4]. Using the microscopic approach the biochemical remission rate for macro GH-PAs is only 40% [10]. Most authors report disease control below 40% independent of traditional surgical techniques if the GH-PA invades the cavernous sinus [2].

Historically, surgical approaches have been confined to the intra- and suprasellar regions to avoid the risk of neurovascular complications associated with cavernous sinus entry. In the late 2010’s and early 2020’s, a few centers began reporting transsphenoidal pituitary surgery where the medial wall of the cavernous sinus was included in the resection [6, 17]. Traditionally, Knosp Grades 3 and 4 have been regarded as adenomas with cavernous sinus infiltration. However, histopathological examinations of a broad range of hormone producing PA specimens have revealed that also low Knosp grade (0–2) adenomas particularly GH-PAs have invaded the medial wall of the cavernous sinus [15]. Studies where surgical resection of GH-PAs have included the medial wall of the cavernous sinus have demonstrated promising biochemical remission rates, ranging from 70 to 90%, depending on surgical expertise and the extent of tumor invasion [6, 15, 17].

Primary complications of GH-PA surgery include cerebrospinal fluid (CSF) rhinorrhea, epistaxis, and postoperative infection. Less common but serious events include pituitary apoplexy, visual loss, and injury to the internal carotid artery (ICA) or cranial nerves (CN)—risks that are particularly relevant during medial cavernous sinus wall resection due to proximity to the ICA and abducens nerve [17, 20]. Postoperative disturbances of ADH secretion and anterior pituitary function may also occur, presenting as syndrome of inappropriate antidiuretic hormone secretion (SIADH), diabetes insipidus, or hypopituitarism, with a risk of life-threatening adrenal insufficiency [13, 20].

It is debated whether more extensive resection increases remission rates in GH-PA and whether the surgical risk is acceptable. In this study, we present our skull base center’s initial experience with extended endoscopic transsphenoidal resection of GH-PAs, including routine medial cavernous sinus wall resection.

## Method

From April 2023 and forward we began removing the medial wall of the cavernous sinus in all patients undergoing transsphenoidal surgery for GH-PAs in our institution. Tumor extent was routinely classified according to cavernous sinus invasion using Knosp grading system [12]. We excised the medial wall on the side adjacent to the adenoma. The cohort, endoscopic resection of adenoma with excision of medial cavernous sinus wall (ERA + MCS), reported in this paper, include our initial 14 patients following primary surgery. Data was extracted from electronic patient records and stored in a RCQP (regional clinical quality program) database for subsequent analysis.

The primary outcome was radiologic and biochemical remission at 3 and 6 months. Biochemical remission was defined as a basal GH concentration < 1 µg/L in combination with a normal age-stratified IGF-1 level, in accordance with the criteria described by *Clemmons *et al*.* [5]. When results were unclear, an oral glucose tolerance test (OGTT) was performed; a nadir GH < 0.4 µg/L on OGTT was considered definitive of biochemical remission. These thresholds were selected based on current consensus guidelines, as outlined by *Melmed *et al*.* [14]. Radiologic remission was defined as the absence of visible residual tumor on the 3-month postoperative MRI as assessed by a neuroradiologist. The ERA + MCS cohort was compared with two historical cohorts treated with traditional surgical techniques: a microscopic resection of adenoma group (MRA) and an endoscopic resection of adenoma group without medial cavernous sinus wall resection (ERA–MCS). Secondary outcomes included intraoperative and postoperative complications using Clavien–Dindo classification. Adenomas were routinely sent for histological evaluation. The medial wall of the cavernous sinus was inconsistently evaluated histologically.

### Statistics

Continuous variables were assessed for normality using the Shapiro–Wilk test. Normally distributed variables were compared across cohorts using one-way analysis of variance (ANOVA), whereas non-normally distributed variables were analyzed using the Kruskal–Wallis test. Categorical variables were compared using Pearson’s χ^2^ test. When an overall group difference was detected, pairwise comparisons were performed. A two-sided p-value < 0.05 was considered statistically significant. Statistical analyses were performed using the software SPSS.

### Surgical technique

**Fig. 1:**
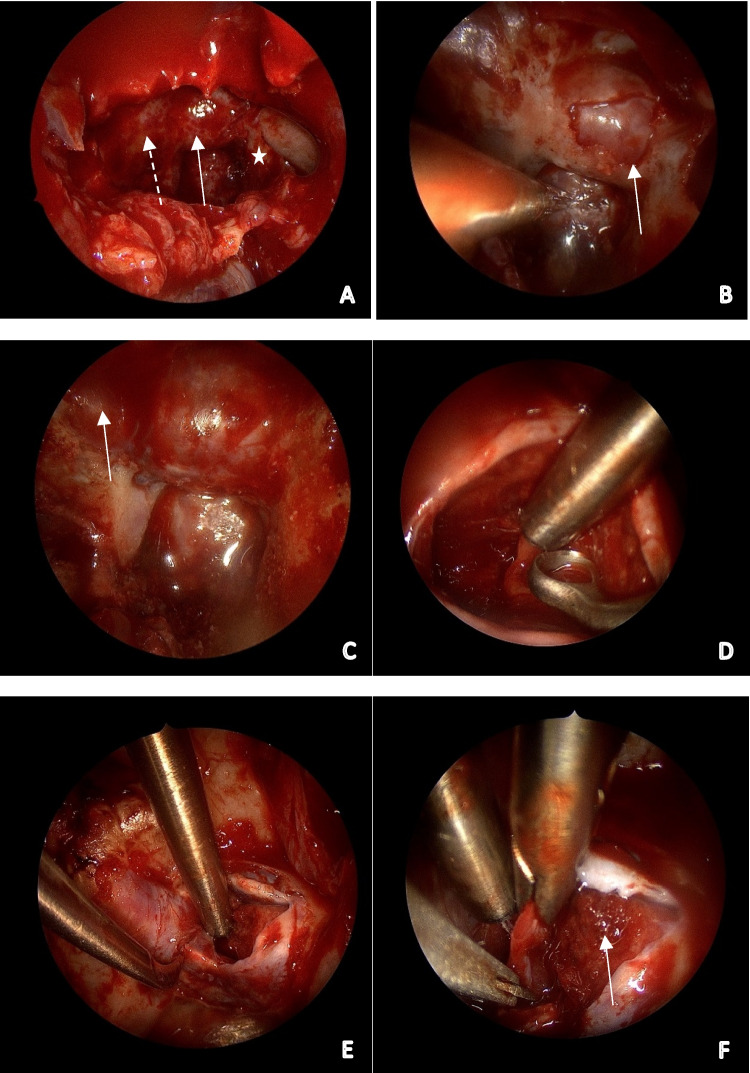
**A** Broad opening of the sphenoid sinus. The sellar floor overlying the pituitary gland is visible (white arrow), along with the internal carotid artery (dotted arrow). A sphenoid septum (marked with a star) is attached to the bone overlying the left internal carotid artery (ICA). Careful drilling is essential when removing this bony septum to avoid tearing into the artery. **B** The sellar floor has been drilled and punched out, revealing the dura-covered pituitary adenoma (white arrow). The suction catheter tip in the clival recess. **C** Removal of bone over the right cavernous sinus and ICA (white arrow). **D** Tumor resection performed using a curette and suction after opening of the dura overlying the adenoma and the pituitary gland (**E**) Opening of the left cavernous sinus medial to the ICA with a sickle-shaped knife. The location of the ICA is verified before opening using micro-Doppler (not in picture). **F** Resection of the medial wall of the cavernous sinus with micro-scissors after mobilization. Note the application of Surgiflo hemostatic agent in the resection area (white arrow)

All procedures utilized an extended endoscopic approach with CT/MR image guidance (Stealth®, Storz inc), performed by our specialized skull base team comprising one or two neurosurgeons and an otolaryngologist. Just before surgery 1500 mg of cefuroxime and 100 mg of Solu-Cortef were administered IV and binasal decongestant with Moffett’s solution was applied. Then a pedicled nasoseptal flap was harvested, preserving the sphenopalatine artery in the pedicle. Thereafter the middle turbinate was either lateralized or if needed partially removed. This was followed by detachment of the nasal septum from the sphenoid rostrum, removal of the sphenoid rostrum, anterior wall and any sphenoid septa if present. Before opening of the sellar floor, a posterior septectomy was conducted enabling four-handed binostril endoscopic technique. The bony impressions including the hypophyseal fossa, ICA, and optic nerves were assessed (Fig. 1A). The bony sellar floor, the anterior sellar wall and the bone overlying the ICA on the side of planned cavernous sinus medial wall resection was thinned with a diamond burr and then removed with punching forceps (Fig. 1B). The position of the ICA was confirmed consecutively using a micro-doppler probe. The exposed dura over the adenoma was then cross-incised with a sickle-shaped knife, allowing careful tumor resection (Fig. 1C + D + E). After removal of all visible adenoma tissue, the adjacent cavernous sinus was opened medial to the ICA and thrombosed with Surgiflo®. Ligaments to the ICA were detached followed by excision of the medial wall with cutting micro scissors (Fig. 1F). Then, the bony and dural defect were sealed with the nasoseptal flap and packed with self-absorbing pads (Naso-pore®) in the sphenoid sinus. Finally, a Merocel nasal pack was inserted on the side of the nasoseptal flap.

### Post-operative treatment and follow-up

Following surgery, patients were transferred to the neuro-intensive care unit for close monitoring until the next day. If no epistaxis was present, the Merocel nasal packing was removed, and the patient was transferred to the department of endocrinology for another 2–4 days observation focused on development of SIADH and diabetes insipidus. All patients received hydrocortisone 50 mg twice a day on day 1. This reduced to 10 + 10 + 5 mg a day before discharge.

Biochemical monitoring was carried out 3 and 6 months post-surgery. At 6 weeks, a Synacthen test was performed, assessing insufficiency in the ACTH-cortisol axis. In addition, hydrocortisone withdrawal was sometimes initiated to evaluate the recovery of endogenous cortisol production. The 3-month follow-up included besides biochemical testing also a postoperative MRI to assess for any evidence of residual tumor or recurrence.

## Results

### Cohort following resection of the medial cavernous sinus wall

**Fig. 2:**
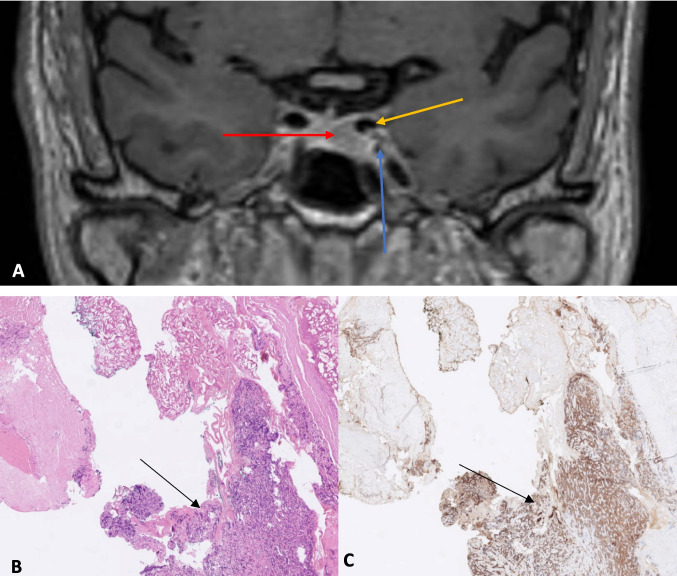
MRI and histological evaluation of resected medial cavernous sinus wall from patient #13. **A** Gadolinium contrast-enhanced T1-weighted MRI. Tumor measuring 8 mm in longest diameter, Knosp grade 0. Red arrow: Adenoma, blue arrow: medial cavernous sinus wall, yellow arrow; intracavernous internal carotid artery. **B**, **C** Hematoxylin & eosin-stained section showed medial wall of the sinus cavernous (black arrows) with crush artifact due to distortion of tissue at removal. The somatotropic cells are invading the small venous spaces separated by fibrous trabeculae. **C** The somatotropic cells show strongly immunopositivity for growth hormone stain

In the study period, 14 patients underwent primary GH-PA resection with excision of the medial cavernous sinus wall. In all cases the Knosp grade was 0–2. The histopathologic examination confirmed the diagnoses. In four patients the excised medial cavernous sinus wall was examined histologically for tumor-invasion. This was observed in 75% (3/4) of the cases (exemplified in Fig. 2).

No patients had visual impairment, and ophthalmological examination demonstrated preserved visual acuity prior to surgery in all cases. All patients were generally healthy, although 10 had sleep apnea as seen in many acromegaly patients. Two patients tested negative for sleep apnea, while the remaining two patients were never tested. One had a history of cardiac disease, paroxysmal atrial fibrillation, with a DC cardioversion performed one year earlier. This patient was not on anticoagulant therapy but had a slightly reduced ejection fraction, as assessed by echocardiography. Four patients had non-insulin-dependent diabetes mellitus, while 6 patients had body-mass index above 30. The side of adenoma location were evenly distributed. In all cases, the nasoseptal flap was harvested from the right.

**Fig. 3:**
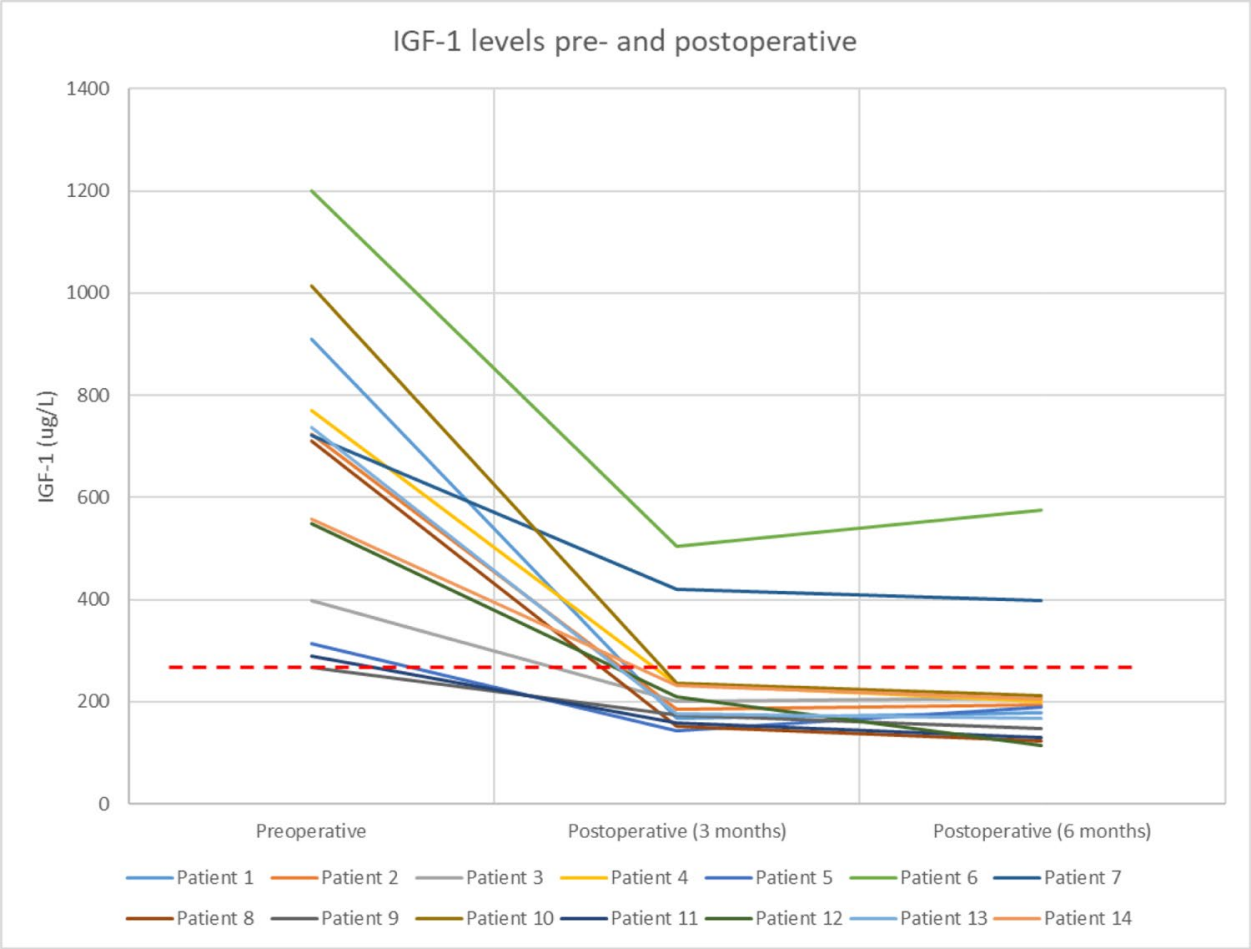
Age-stratified Insulin-like growth factor 1 (IGF-1) in acromegaly patient at baseline, 3 and 6 months postoperative following ERA + MCS

For 12/14 (86%) patients with a newly diagnosed GH-PA undergoing endoscopic resection, including the medial wall of the cavernous sinus, normalization of the GH-axis was observed 3 and 6 months postoperatively (Table 1, Fig. 3). Radiological 3-months follow-up MRI was without remnant tumor in all cases where biochemical remission was achieved. Additionally, no residual tumor was evident on MRI in one of the two patients not being in biochemical remission.

Complications were graded according to the Clavien–Dindo classification. No grade III–V complications occurred (Table 1). Self-limiting epistaxis and serosanguinous nasal drainage were classified as grade I complications. Two patients developed delayed transient SIADH, managed with fluid restriction and sodium correction (grade II). One patient experienced a secondary adrenal crisis requiring high-dose intravenous hydrocortisone. The patient was admitted to a local hospital ward and subsequently transferred to a tertiary endocrine unit but did not require intensive care management; this event was therefore classified as grade II. No ICA or CN injuries were observed.

**Table 1:** Head-to-head comparison of surgical characteristics, remission rates, and complications

Parameter	MRA	ERA-MCS	ERA + MCS	Statistics
Cases (number)	29	16	14	
Age (mean)	51	50	51	*P* = *0.97*
Male/Female	15/14	10/6	7/7	*P* = *0.11*
Surgical duration (mean)	110 min	69 min	178 min	*P* = *7,3* × *10*^*–14*^
Largest tumor diameter (median)	16 mm	11 mm	12 mm	*P* = *0.0033*
Knosp grade				*P* = *0.085*
0	6	2	4	
1	14	9	6	
2	9	5	4	
Preoperative IGF1 (median)	823 ug/L	664 ug/L	716 ug/L	*P* = *0.02*
Postoperative IGF (median)	359 ug/L	248 ug/L	194 ug/L	*P* = *0.052*
Preoperative GH (median)	19.7 ug/L	5.4 ug/L	2.7 ug/L	*P* = *0.0016*
Suppression on OGTT^a^	9	6	10*	*P* = *1.3* × *10*^*–6*^
Biochemical remission*	31% (9/29)	38% (6/16)	86% (12/14)	*P* = *0.0025*
Radiological remission	38% (11/29)	44% (7/16)	93% (13/14)	*P* = *0.0024*
Complications				
*Clavien–Dindo grade*				
*I*				
Epistaxis	Unknown	Unknown	3	
Rhinoliquorrhea	0	0	0	
*Clavien–Dindo grade*				
*II*				
Rhinoliqourrhea	6	2	0	
Epistaxis	1	1	1	
SIADH	2	1	2	
Meningitis	2	1	0	
Sec. adrenal crisis	0	0	1	
Diabetes insipidus	2	1	0	
*Clavien–Dindo grade*				
*III-IV*				
Visual loss	1	0	0	
ICA injury	0	0	0	
Meningitis	0	0	0	
Sec. adrenal crisis	0	0	0	

*MRA* microscopic resection of adenoma, *ERA–MCS* endoscopic resection of adenoma without resection of the medial cavernous sinus wall, *ERA* + *MCS* endoscopic resection of adenoma with resection of the medial cavernous sinus wall

a) Oral glucose tolerance test (OGTT): Postoperative nadir GH < 0.4 µg/L was considered as biochemical remission [5]. *Patients with postoperative IGF1 within the age-stratified normal range and baseline GH < 0.4 µg/L were also considered as biochemical remission [5]

### Comparison with historical cohorts

Table 1 presents a head-to-head comparison of the three surgical cohorts MRA, ERA–MCS and ERA + MCS. The cohorts were comparable with respect to age and gender distribution. The MRA group, however, tended to have slightly larger adenomas and a higher proportion of patients with Knosp grade 2 GH-PAs.

Biochemical remission differed significantly across the cohorts, with the ERA + MCS technique achieving the highest remission rate (86%), outperforming both microscopic and endoscopic without medial sinus wall resection approaches. Surgical duration was somewhat longer in the ERA + MCS cohort, primarily due to the routine of harvesting a nasoseptal flap.

The complication rate for endoscopic procedures was generally low, with only a few cases of SIADH and rhinorrhea. In contrast, the microscopic technique was associated with a higher complication rate, particularly postoperative CSF rhinorrhea.

Figure 3 illustrates the reduction in age-stratified IGF-1 levels, demonstrating its value as a predictive marker of biochemical remission.

## Discussion

### Comparison with existing studies

The results of our study aligned with findings in the existing literature, demonstrating that resection of the medial wall of the cavernous sinus is both effective and safe for GH-PAs. Complete biochemical remission was achieved in 86% of primary GH-PA cases (Knosp grade 0–2), which is consistent with biochemical remission rates reported in other studies of similar surgical approaches. Pontes et al*.* (2023) found biochemical remission rates of 70% for cases with medial sinus wall resections, though outcomes were closely linked to the extent of tumor invasion and surgical expertise (7). A more recent study by Ling Tian et al*.* also compared the efficacy of medial cavernous sinus wall resection at their institution and similarly reported higher remission rates favoring medial wall removal (86% vs 67%). The difference was even more pronounced in the Knosp grade 3–4 subgroup, with remission rates of 78% compared with 31% [21]. The Mohyeldin group reported a biochemical remission rate of 92% in their sub-cohort of GH-PAs. Their study primarily included smaller GH-PAs (Knosp Grade 0–2) and incorporated histological analysis, which confirmed that even small adenomas exhibited a propensity for invading the medial wall of the cavernous sinus—an especially notable characteristic of GH-PAs. In GH-PA, they found that 25% of Knosp 0, 67% of Knosp 1, and 100% of Knosp 2 tumors had tumor invasion of the medial cavernous sinus wall [15]. Our results align closely with the Mohyeldin group, most likely due to the similar Knosp grades included in our study. Another recent single-center evaluation by Oberman et al*.* has also shown promising biochemical remission rates. In GH-PA, the biochemical remission rate in this study was 88.5% when the resection included the medial cavernous sinus wall. In these cases, invasion of the medial cavernous sinus wall was confirmed histological even in smaller GH-PAs (12). Generally, the number of patients in the existing literature is low, but when comparing the above reported remission rates on medial cavernous sinus wall resection to results on GH-PA without medial cavernous sinus wall resection the results stand out. In a review by Campbell et al*.* in 2010 biochemical remission rates of 54.5% were reported when endoscopic resection included only the adenoma and not the adjacent structures [4]. Similarly, Shirvani et al*.* conducted a single-center study of 130 prospective cases and found an overall biochemical remission rate of 60.5%, including both micro and macro GH-PA cases. Notably, they reported a lack of biochemical remission when invasion of the cavernous sinus was evident [19]. The remission rates in our historical cohorts were even lower being 31% in the microscopic cohort and 38% in the endoscopic cohort without medial cavernous sinus wall resection.

### Postoperative complications

Two cases of SIADH (14%) were observed and both resolved with fluid restriction. Larger cohort studies estimate the incidence of SIADH following pituitary surgery to be 5–10% SIADH (6). The higher rate observed in our study is most likely due to the small sample size, representing a statistical anomaly. To our knowledge, no literature specifically addresses the occurrence of SIADH following resection of the medial cavernous sinus wall. Previous studies by Yuen et al*.* and Deaver et al*.* have documented SIADH and hyponatremia as potential postoperative complications of pituitary surgery. Notably, these studies also demonstrated that implementing postoperative water restriction protocols can reduce the incidence of SIADH by two- to threefold [9, 24].

Most importantly, no major complications such as ICA injury, CN deficits, or postoperative neuro infection were observed. Additionally, neither intraoperative nor postoperative CSF leakage was observed. The literature suggests an ICA injury risk of approximately 0.5%, with an even lower incidence of optic nerve damage or abducens nerve injury leading to diplopia, further supporting the safety of the extended surgical approach [6, 17]. A 2019 systematic review and meta-analysis assessing the safety of medial wall resection reported no significant difference in major complications when comparing traditional endoscopic pituitary resection with approaches that include resection of the medial cavernous sinus wall [17, 20]

Our findings highlight the importance of meticulous surgical planning and execution. The use of micro-doppler probes, multidisciplinary team experience and careful hemostatic control are without question important factors in avoiding these very serious intraoperative complications.

### Perspectives and future directions

This study adds to the growing body of evidence indicating superior remission rates when routinely including resection of the medial cavernous sinus wall in the treatment of GH-PA compared to adenoma resection alone. As our initial cohort only includes primary cases with Knosp grades 0–2 its applicability to higher Knosp graded adenomas (3–4) and recurrent cases remain uncertain based on our data. Recurrent cases as well as cases with adenoma tissue lateral to the ICA (Knosp grad 3–4) have an increased risk profile in relation to ICA injury, CN deficits, rhinoliquorrhea and meningitis.

### Strengths and limitations

The strengths of this study are the Knosp grade matched head-to-head comparison of three different transsphenoidal surgical procedures in the treatment of low Knosp grade GH-PA; the former widely used microscopic adenoma resection, the current widely used endoscopic adenoma resection without medial cavernous sinus wall and the currently limited used endoscopic adenoma resection with routinely medial cavernous sinus wall removal. The study is a single-center study where all surgeries have been largely carried out by the same dedicated team of three neurosurgeons (FRP, CBP, BH) during all the years. This strengthens the value of the procedure comparisons despite the retrospective nature of the study. The lack of routine histological evaluation of the resected cavernous sinus walls is a limitation. This practice has therefore been changed, and all resected walls are now histologically evaluated for the benefit of future research. Other limitations are the relatively low sample size, the inclusion of only Knosp grade 0–2 adenomas and only primary cases. Future studies should include larger cohorts, higher Knosp graded adenomas and both primary and recurrent cases to assess the broader applicability of the technique.

## Conclusion

Our study demonstrated that transsphenoidal endoscopic resection of Knosp grade 0–2 GH-PA, with medial cavernous sinus wall resection resulted in 86% remission rate, evaluated at both 3 and 6 months. A remission rate of 86% was significantly better than remission rates of 31% and 38%, respectively, after transsphenoidal microscopic or endoscopic resection of adenoma without medial cavernous sinus wall removal. Moreover, the new procedure was safe with no severe complications.

## Data Availability

The data are available upon request.
